# Predicting Single‐Cell Perturbation Responses Across Biological Contexts With a Deep Generative Model Integrating Optimal Transport

**DOI:** 10.1002/advs.77461

**Published:** 2026-08-29

**Authors:** Jialiang Wang, Ziqi Liu, Zhengqian Zhang, Yikun Cao, Junjun Ren, Peng Cheng, Jingjing Tian, Lingyun Xie, Xin Lu, Zhanwei Du, Yongzhuang Liu

**Affiliations:** ^1^ School of Computer Science and Technology Harbin Institute of Technology Harbin China; ^2^ HIT‐HULU Joint Institute of Industrial Technology in Precision Medicine Harbin China; ^3^ Genvista Technology Co., Ltd. Harbin China; ^4^ College of Systems Engineering National University of Defense Technology Changsha China; ^5^ School of Public Health and Emergency Management Southern University of Science and Technology Shenzhen China; ^6^ WHO Collaborating Centre for Infectious Disease Epidemiology and Control School of Public Health Li Ka Shing Faculty of Medicine The University of Hong Kong Hong Kong SAR China

**Keywords:** bioinformatics, computational biology, drug discovery, generative model, machine learning, modelling biological systems

## Abstract

Predicting how single cells respond to perturbations is a central problem in computational biology, with potential relevance to emerging artificial intelligence virtual cell (AIVC) research and drug‐discovery efforts. However, substantial variation in perturbation responses across biological contexts and the limited generalizability of current models make prediction across cell types, patients, species, and other contexts particularly challenging. To address this challenge, we present single‐cell perturbation inference via latent optimal transport (scPILOT), a query‐conditioned framework for transferring responses to previously observed perturbations across biological contexts. scPILOT learns a generative latent representation through discriminator‐assisted training and separates perturbation inference into cell‐level response estimation from observed contexts and query‐specific response transfer using latent optimal transport. Across held‐out cell‐type, patient, and species benchmarks, scPILOT achieved context‐averaged *R* ^2^_mean_/MMD^2^ values of 0.945/0.137, 0.598/0.025, and 0.853/0.287, respectively. It also maintained strong population‐average accuracy in a held‐out cell‐line benchmark, while complementary analyses indicated that performance was associated with dataset learnability and query–context match. With the continued expansion of single‐cell perturbation datasets, scPILOT may provide a practical framework for transferring responses to previously observed perturbations across increasingly diverse biological contexts.

## Graphical abstract

Single‐cell perturbation responses are predicted across held‐out biological contexts using scPILOT, a query‐conditioned two‐stage latent response‐transfer framework. A shared latent representation supports cell‐level response estimation by latent optimal transport, followed by Leiden‐localized query‐specific transfer and adaptive weighting. The resulting predictions closely match observed mean expression and single‐cell distributions across cell types, patients, species, and cell lines.

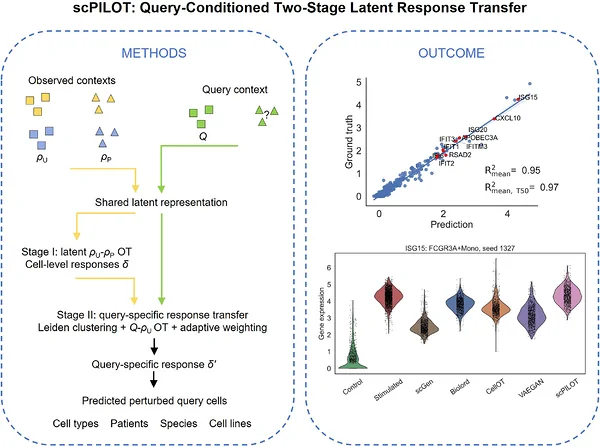

## 1 Introduction

Recent advances in single‐cell sequencing technologies have enabled the generation of high‐resolution molecular profiles that capture genomic, transcriptomic, epigenomic, and proteomic information from individual cells [1]. These advances make it possible to characterize cellular responses to perturbations at single‐cell resolution [2, 3]. However, single‐cell sequencing can be very expensive and time‐consuming due to vast combinatorial spaces caused by the high degree of perturbation heterogeneity [4] and the complicated situations of cells [5]. High‐throughput screening is infeasible or practically/ethically prohibitive in some situations due to technical constraints [6] or ethical considerations [7], such as when researching dense connective tissues (e.g., cartilage, tendon, and bone), where the abundance of extracellular matrix and sparsity of cells make the preparation of high‐quality cell suspensions particularly challenging, and in studies involving sensitive personal biological information, where stringent requirements for privacy and data security must be upheld.

With the rapid expansion of accessible single‐cell sequencing data, AI‐driven learning and prediction of cellular perturbation responses is rapidly emerging as a mainstream approach [8]. In silico perturbation prediction enables systematic exploration of vast hypothesis spaces and may reduce reliance on experimental single‐cell profiling in settings where such measurements are technically infeasible or practically/ethically constrained. Recent perspectives have identified these capabilities as important elements in the development of artificial intelligence virtual cells (AIVCs) [9], which are envisioned to simulate and predict cellular behaviours and responses with high fidelity, potentially complementing or partially substituting for conventional experimental approaches and ultimately facilitating drug discovery. To help realize this broader potential, efficient computational methods that leverage single‐cell sequencing data are needed to predict cellular perturbation responses accurately and at scale. Such methods can systematically explore large hypothesis spaces, prioritize candidate hypotheses, and guide targeted experimental validation. With continued improvements in predictive accuracy and generalizability, computational perturbation models are envisioned to complement experimental studies and potentially reduce or replace selected experiments, particularly where direct measurements are technically infeasible or practically/ethically constrained. These capabilities may improve the efficiency of experimental research and contribute to broader efforts in virtual‐cell modelling and drug discovery.

To date, many deep learning models exist for single‐cell perturbation prediction [4, 10, 11, 12, 13, 14]. They generally follow a similar architecture: an autoencoder (AE) and latent space manipulations. An AE is a neural network architecture that typically consists of an encoder and a generator (a decoder). AEs have two capabilities: feature extraction and data generation. For single‐cell sequencing data, a cell is a sample, and the gene expression levels are considered features. Since the gene number is too large to characterize a cell efficiently, an encoder is needed to encode a cell into a low‐dimensional latent vector. Then, manipulations that represent a specific kind of perturbation are performed on this latent vector. Finally, the vector is input into a generator to obtain what this cell is probably like after responding to the perturbation.

These models typically operate under the assumption that the responses to perturbations can be captured at the cell group level, where unperturbed and perturbed cells are matched on the basis of group characteristics, such as cell type. However, this assumption fails to account for the heterogeneity of individual cells within a group. A major limitation arises from the inherent constraints of single‐cell sequencing technologies, where each cell is usually fixed, stained, or destroyed during the measurement process [12, 15, 16], preventing direct pairing of unperturbed and perturbed states for the same cell. As a result, perturbation effects are often estimated as the average difference between the latent vectors of unperturbed and perturbed cells within the same group. This oversimplifies the variability of individual cell responses and undermines the ability of the models to generalize across different biological contexts, leading to potential performance degradation when applied to new data.

This issue also arises in single‐cell perturbation analysis, where optimal transport (OT) has been used to infer correspondences between unperturbed and perturbed cell populations. CINEMA‐OT reduces the dimensionality of scRNA‐seq data and uses OT to infer cell‐level couplings for downstream perturbation analysis [16]. CellOT further extends OT to generative prediction by learning a neural transport map between unperturbed and perturbed populations [12]. However, estimating a control‐to‐perturbed mapping within observed biological contexts does not by itself determine how heterogeneous perturbation responses should be assigned to cells in a held‐out context for which only the unperturbed state is available. Cross‐context prediction therefore requires two related but distinct operations: estimating cell‐level perturbation responses from observed control–perturbed populations and transferring these responses to the unperturbed query population according to similarities in baseline cellular state.

To address this gap, we introduce single‐cell perturbation inference via latent optimal transport (scPILOT), a query‐conditioned framework for transferring responses to previously observed perturbations across biological contexts. scPILOT learns a generative latent representation through discriminator‐assisted training and separates perturbation inference into cell‐level response estimation from observed contexts and query‐specific response transfer using latent OT. Leiden [17]‐based localization and adaptive weighting determine how response estimates from multiple observed populations are transferred and integrated for the query population. We evaluate scPILOT across held‐out cell‐type, patient, species, and cell‐line benchmarks, together with component‐wise ablations and analyses of dataset learnability and query‐to‐observed‐context mismatch. These experiments characterize scPILOT as a response‐transfer approach for held‐out biological contexts in which unperturbed query cells are available.

## 2 Results

### 2.1 scPILOT for Query‐Conditioned Perturbation Prediction Through Two‐Stage Latent Response Transfer

**FIGURE 1:**
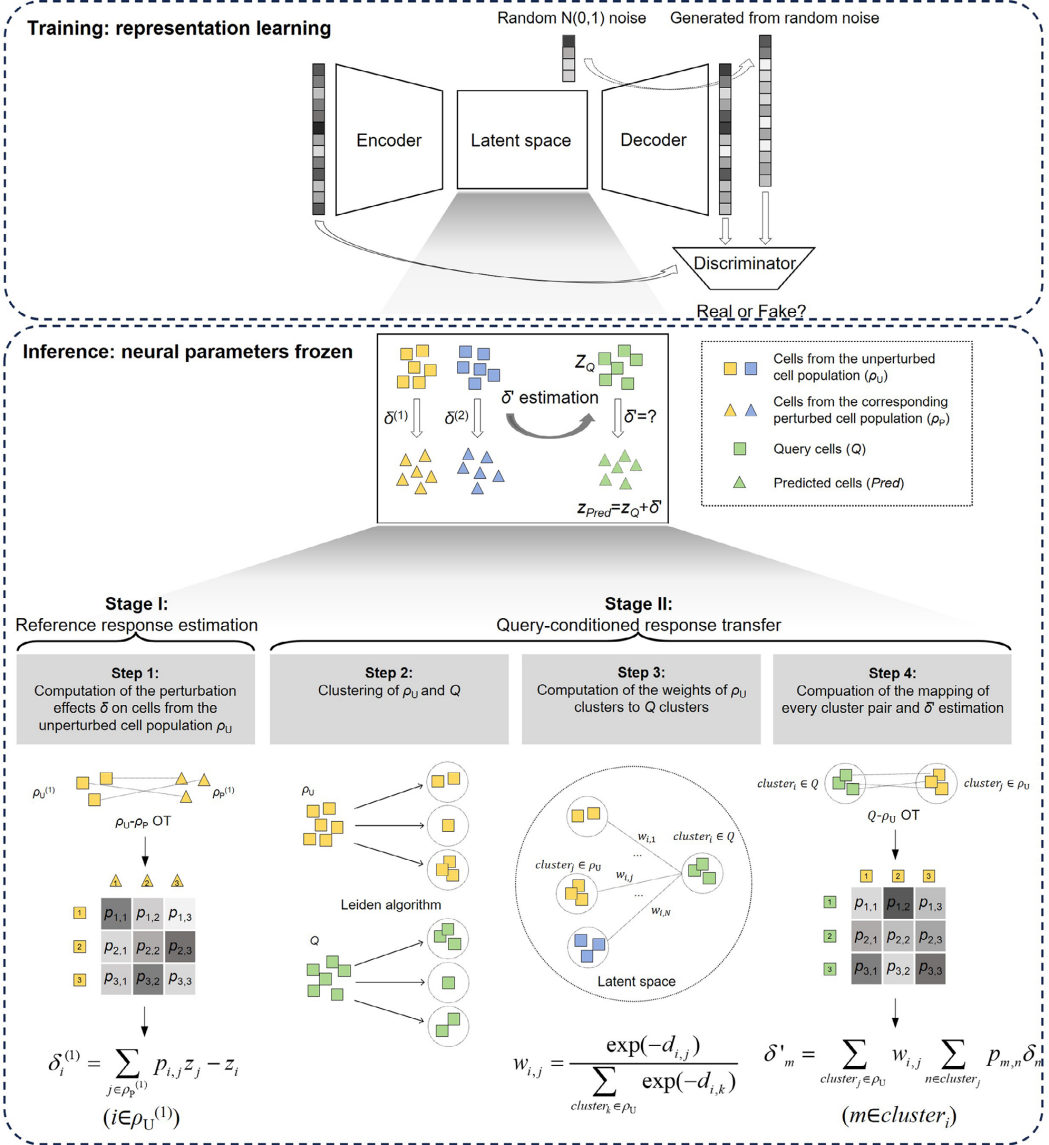
scPILOT performs query‐conditioned perturbation prediction through two‐stage latent response transfer. During training, the encoder, generator, and discriminator are optimized to learn a generative latent representation of the observed cellular states. During inference, the learned neural‐network parameters are frozen. In the first transport stage, *ρ*_U_–*ρ*_P_ OT estimates heterogeneous cell‐level response vectors. In the second stage, Leiden clustering defines local query and reference neighbourhoods, *Q*–*ρ*_U_ OT transfers the reference response vectors to individual query cells, and OT‐cost‐based adaptive weighting integrates estimates from multiple *ρ*_U_ clusters. The resulting query‐specific response vectors are added to the query latent states and decoded to obtain the predicted perturbed expression profiles. (*z* denotes latent vectors, *δ* denotes the response vectors of cells from the unperturbed cell population *ρ*_U_, *δ'* denotes the estimated response vectors of the query cells *Q*, *p* denotes the elements in the OT mapping matrices, *d* denotes the squared 2‐Wasserstein distance, and *w* denotes the weights of *ρ*_U_ clusters to *Q* clusters).

scPILOT comprises a generative representation‐learning stage and a query‐conditioned inference procedure (Figure 1). During training, the encoder, generator, and discriminator learn a latent representation of the observed cellular states. During inference, the learned neural‐network parameters are fixed, and latent OT is used first to estimate heterogeneous perturbation responses from observed unperturbed and perturbed populations and subsequently to transfer these responses to unperturbed query cells. Leiden‐based localization and adaptive weighting determine how response estimates from multiple observed populations are transferred and integrated for the query population.

**During representation learning,** the encoder reduces the dimensionality of a cell, where the features represent genes, causing the cell to approach a standard normal distribution by using Kullback–Leibler (KL) divergence. The generator can generate a cell from a standard normally distributed random noise so that it can reconstruct the input. The discriminator classifies a cell as real or fake so that it can act as an adversary to the generator and thus make the generator generate cells that are more similar to the input. Cell *i* of group *t* and condition *c* (0 for unperturbed or 1 for perturbed) is denoted by *x_i,t,c_*. In the latent space, cell *i* becomes *z_i,t,c_* via the encoder. Then, it is input into the generator and becomes ${\hat{x}}_{i,t,c}$. After ideal training, *x_i,t,c_* and ${\hat{x}}_{i,t,c}$ should be the same. We also sample a set of random noise *z_p_* whose number of elements equals the number of inputs and input those values into the generator to obtain ${\hat{x}}_{p}$. We then train the discriminator to correctly classify the input cells as either real (*x*) or generated ($\hat{x}$, ${\hat{x}}_{p}$). Simultaneously, the generator is optimized to produce synthetic cells ($\hat{x}$, ${\hat{x}}_{p}$) that are indistinguishable from real cells, thereby fooling the discriminator.

During the inference stage, a group of query cells only under unperturbed conditions and several groups of cells under both unperturbed and perturbed conditions are input into the encoder. In the latent space, we match cells from the unperturbed cell population *ρ*_U_ and the corresponding perturbed cell population *ρ*_P_ into pairs and then approximate the query cells *Q* with *ρ*_U_ by means of clustering and OT to estimate the perturbation effects on *Q*. The latent manipulations in scPILOT consist of 4 main steps. Step 1 computes the perturbation effects *δ* on *ρ*_U_ with *ρ*_U_–*ρ*_P_ OT, which maps every cell group of *ρ*_U_ to *ρ*_P_, and Step 2 divides *ρ*_U_ and *Q* into smaller clusters with the Leiden algorithm [17]. Any combination of an *ρ*_U_ cluster and a *Q* cluster forms a cluster pair. Step 3 computes the weights of the *ρ*_U_ clusters to every *Q* cluster according to the squared 2‐Wasserstein distance with softmax, and Step 4 computes the mapping of every cluster pair with *Q*–*ρ*_U_ OT and estimates the perturbation effects *δ'* on *Q* according to the final weights obtained in Steps 3 and 4. Eventually, we add the estimated *δ'* to *Q* to obtain the predicted cells (*Pred*) and input them into the generator to obtain counterfactual prediction.

Through this procedure, scPILOT first estimates heterogeneous cell‐level perturbation responses from observed populations and then performs query‐conditioned transfer and integration of these responses for the held‐out population. To demonstrate the advantage of scPILOT in terms of predicting single‐cell perturbation responses, we benchmarked it against scGen [11], CellOT [12], biolord [14], VAEGAN, and a baseline with unchanged inputs called identity that outputs the same cells that were input without any processing.

### 2.2 Performance of scPILOT and State‐of‐the‐Art Models for Single‐Cell Perturbation Prediction

**FIGURE 2:**
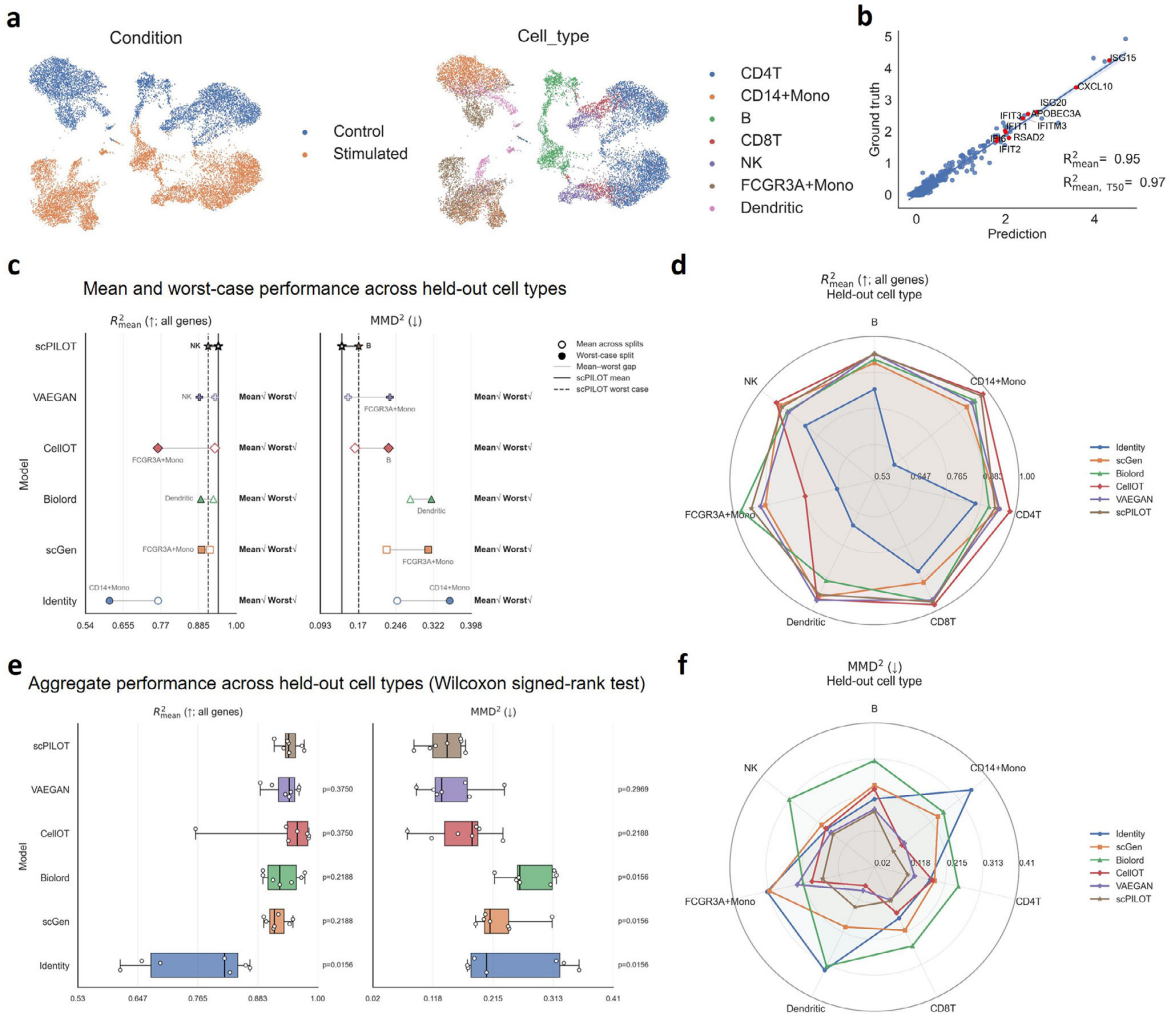
scPILOT achieves strong and robust perturbation prediction across held‐out cell types. (a) UMAP visualization of the dataset for predicting IFN‐β‐stimulated cells. We used a dataset of *n* = 18,848 PBMCs provided by Kang et al. [18], labeled by condition and cell type. (b) Linear regression of mean gene expression between predicted and perturbed FCGR3A+Mono cells for seed 1327, with the top 10 DEGs highlighted (*n*_genes_ = 6,998; *R* ^2^ denotes squared Pearson correlation). (c) Mean and empirical worst‐case *R* ^2^_mean_ and MMD^2^ across the seven held‐out cell types. (d, f) Cell‐type‐specific *R* ^2^_mean_ and MMD^2^, respectively. (e) Aggregate performance across held‐out cell types and paired comparisons between scPILOT and each baseline. For (c–f) each metric was first averaged across three random seeds for each held‐out cell type. The *p* values in **e** were obtained using two‐sided paired Wilcoxon signed‐rank tests across the seven held‐out cell types (*n* = 7).

We applied the 6 models to an open‐access single‐cell RNA‐sequencing (scRNA‐seq) dataset of human peripheral blood mononuclear cells (PBMCs), including 7 cell types (B, CD14+Mono, CD4T, CD8T, Dendritic, FCGR3A+Mono, and NK) stimulated with interferon‐beta (IFN‐β), which was provided by Kang et al. [18], in which cell behaviour changes significantly under perturbation. Each cell type was treated in turn as the held‐out query context, with its unperturbed cells available to the model and its perturbed cells reserved as the ground truth for final evaluation. Each held‐out‐cell‐type experiment was evaluated using three predefined random seeds. Figure 2a shows the Uniform Manifold Approximation and Projection (UMAP) [19] visualization of the dataset on this benchmark. We also performed a linear regression between the mean gene expression of the scPILOT‐predicted and perturbed FCGR3A+Mono cells for seed 1327, which showed strong agreement (Figure 2b; for other cell types and seeds, see Figure S1). scPILOT also accurately predicted all of the other cell types (average *R* ^2^_mean_ = 0.945, Figure 2c,d).

To account for variation across model initializations, we first averaged *R* ^2^_mean_ across the three random seeds for each held‐out cell type and then summarized performance across the seven cell‐type prediction tasks. scPILOT achieved the highest context‐averaged *R* ^2^_mean_ (0.945, other best 0.935) and the best empirical worst‐case performance (0.914, other best 0.894) among the evaluated methods (Figure 2c). The cell‐type‐specific results further showed that scPILOT maintained consistently high predictive accuracy across the seven held‐out cell types, although several competing methods achieved comparable performance in individual tasks (Figure 2d). The largest separation was observed for held‐out FCGR3A+Mono cells, for which several baselines, particularly CellOT, showed substantially lower *R* ^2^_mean_. Taken together, these descriptive results indicate that scPILOT provided both strong average accuracy and robust performance on the most challenging held‐out cell type.

To evaluate the agreement between the predicted and observed perturbed cell distributions beyond population‐average expression, we computed the squared maximum mean discrepancy, MMD^2^ [20], using the top 50 differentially expressed genes, with lower values indicating closer distributional agreement (for other metrics, see Methods and Figure S2). After averaging the results across the three random seeds for each held‐out cell type, scPILOT achieved the lowest context‐averaged MMD^2^ (0.137, other best 0.149) and the best empirical worst‐case performance (0.171, other best 0.231) among the evaluated methods (Figure 2c). The cell‐type‐specific results further showed that scPILOT maintained relatively low distributional discrepancies across all seven held‐out cell types, although individual baselines achieved comparable or lower values in some prediction tasks (Figure 2f). Notably, the worst‐case MMD^2^ of scPILOT remained lower than the worst‐case values of all baseline methods, indicating less pronounced performance degradation on the most challenging held‐out cell type. Together, these descriptive results suggest that scPILOT provided both strong average distributional agreement and robust performance across held‐out cell types.

We further performed two‐sided paired Wilcoxon signed‐rank tests using the seven held‐out cell types as independent paired observations after averaging across the three random seeds. For *R* ^2^_mean_, scPILOT significantly outperformed identity mapping, whereas the differences from the other predictive models did not reach statistical significance. For MMD^2^, scPILOT showed significantly better distributional agreement than identity mapping, scGen, and biolord, while the differences from CellOT and VAEGAN were not statistically significant (Figure 2e). Because these context‐level comparisons were necessarily based on only seven independent held‐out cell types, the tests had limited resolution for detecting modest differences among closely performing methods. Nevertheless, the highest context‐averaged *R* ^2^_mean_, the lowest context‐averaged MMD^2^, and the best empirical worst‐case performance for both metrics consistently support the strong overall performance and robustness of scPILOT across held‐out cell types.

**FIGURE 3:**
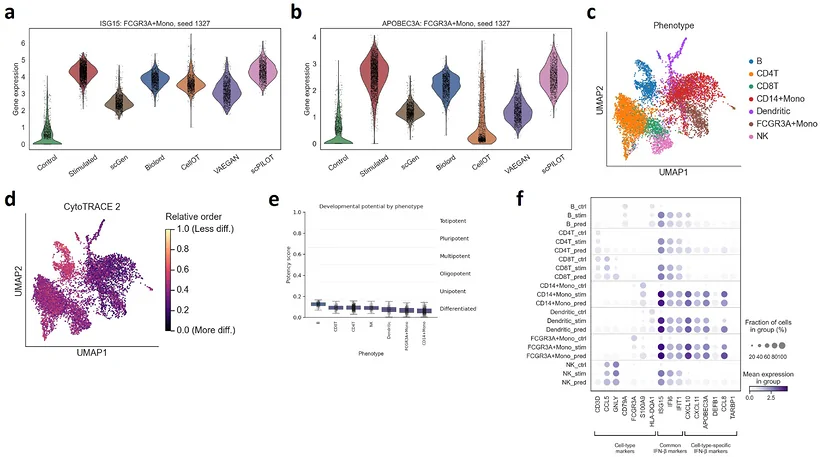
scPILOT provides a good estimation of the full distribution of DEGs and is suitable for downstream differentiation. (a, b) Marginal distributions of ISG15 and APOBEC3A, respectively, for held‐out FCGR3A+Mono cells and seed 1327 (*n*_cells, ctrl_ = *n*_cells, pred_ = 1232, *n*_cells, stim_ = 2790). (c, d) UMAP visualization of CytoTRACE2 [23] analysis, coloured according to cell type and differentiation order. (e) Boxplot of the potency score by CytoTRACE2 [23] (*n*_B_ = 928, *n*_CD8T_ = 643, *n*_CD4T_ = 2715, *n*_NK_ = 571, *n*_Dendritic_ = 670, *n*_FCGR3A+Mono_ = 1232, *n*_CD14+Mono_ = 2184). (f) Dot plot comparing control (ctrl), observed stimulated (stim), and scPILOT‐predicted cells (pred) across the seven held‐out cell types. Predicted cells from the three random seeds were pooled within each held‐out cell type for visualization, whereas the corresponding control and stimulated cells were included once.

In addition to the global metrics, we also obtained the marginal distributions of the top DEGs estimated with scanpy [21], including ISG15, which is reported as the top uniform response gene to IFN‐β by Butler et al. [22], and another interferon‐stimulated gene (ISG), APOBEC3A. We observe that scPILOT provides a good estimation for the full distribution, whereas the others less successfully match the heterogeneous states of the perturbed population (Figure 3a,b; Figure S3).

The immune response of immune cells is closely related to their degree of differentiation [24, 25]. We analysed the developmental potential of the cells with CytoTRACE2 [23] and found that among the PBMCs, FCGR3A+Mono has the lowest potential, indicating that it is at the downstream stage of differentiation (Figure 3c–e). Since scPILOT performs well and steadily, we conclude that it characterizes differentiation details better than the baselines and is suitable for cells downstream of differentiation.

### 2.3 Different Responses of Common Marker Genes Among Cell Types and Cell‐Type‐Specific Marker Genes Characterized by scPILOT

The gene expression changes in a group of perturbed cells may exhibit a specific pattern depending on their biological features, such as individual receptors, signalling pathways, and regulatory networks. Some changes are shared among most cell types, whereas some changes are unique to only one or two cell types. Capturing both types of changes is important for understanding disease progression mechanisms and may help inform drug‐dose selection [26, 27]. As described above, each cell type was treated in turn as the held‐out query context. The resulting predictions recapitulated both shared IFN‐β responses and cell‐type‐specific expression changes across the seven cell types (Figure 3f).

We integrated the gene expression of unperturbed, perturbed, and scPILOT predicted cells on 3 different kinds of marker genes previously reported by Butler et al. [22]. The marker genes include three parts: cell‐type‐specific markers regardless of perturbations, such as CD3D for T cells and CD79A for B cells; IFN‐β markers that show obvious responses under the perturbation of IFN‐β in all cell types, including ISG15, IFI6 and IFIT1; and cell‐type‐specific IFN‐β markers that obviously respond to IFN‐β in one or two cell types, such as CXCL10 for myeloid cells. scPILOT predicts accurate responses of common markers and cell‐type‐specific markers when predicting all cell types in the PBMC dataset provided by Kang et al. [18].

### 2.4 Generalization to Unseen Patients by scPILOT and the Baselines

**FIGURE 4:**
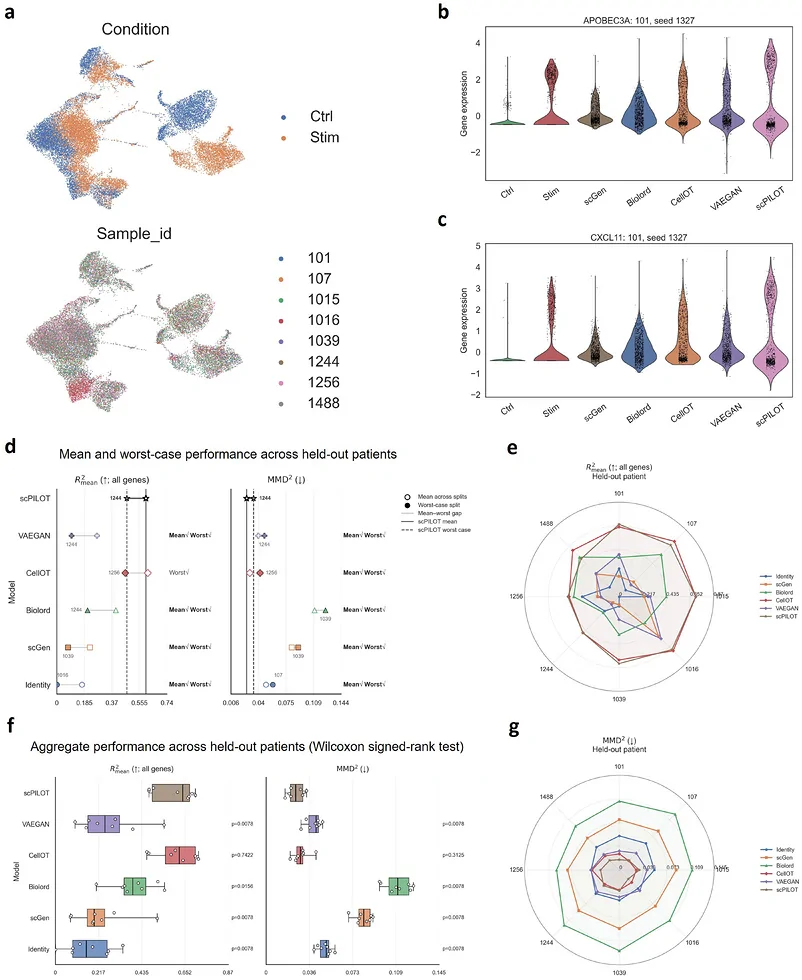
scPILOT shows strong and robust perturbation prediction across held‐out patients. (a) UMAP visualization of the PBMC dataset consisting of eight patients (*n* = 24 483) provided by Kang et al. [18], labeled by condition and patient sample ID. (b, c) Marginal distributions of APOBEC3A and CXCL11, respectively, for held‐out patient 101 and seed 1327 (*n*_cells, ctrl_ = *n*_cells, pred_ = 871, *n*_cells, stim_ = 1128). (d) Mean and empirical worst‐case *R* ^2^_mean_ and MMD^2^ across the eight held‐out patients. (e, g) Patient‐specific *R* ^2^_mean_ and MMD^2^, respectively. (f) Aggregate performance across held‐out patients and paired comparisons between scPILOT and each baseline. For (d–g) each metric was first averaged across three random seeds for each held‐out patient. The *p* values in (f) were obtained using two‐sided paired Wilcoxon signed‐rank tests across the eight held‐out patients (*n* = 8).

Generalization to held‐out patients represents a clinically relevant and challenging setting for single‐cell perturbation prediction because patient‐specific transcriptional profiles may reflect both biological heterogeneity and sample‐associated technical variation. We therefore evaluated scPILOT using the PBMC dataset provided by Kang et al. [18], which was annotated by patient sample ID (Figure 4a). Each of the eight patients was treated in turn as the held‐out query context: the patient's unperturbed cells remained available for representation learning and perturbation inference, whereas the corresponding perturbed cells were withheld and used only as the ground truth for final evaluation. Each held‐out‐patient experiment was repeated using three predefined random seeds.

In contrast to the other models, scPILOT successfully depicts the marginal distributions of marker genes with bimodal shapes (Figure 4b,c; Figure S4). Since the cells of one patient contain all of the PBMC cell types, they usually respond in two completely different ways. For a specific marker gene, some responses are strong, while others show almost no response. Thus, the marginal distributions usually polarize, which is not captured by the other models.

After averaging each metric across the three random seeds within each held‐out patient, scPILOT achieved a context‐averaged *R* ^2^_mean_ of 0.598, comparable to that of CellOT, while showing the best empirical worst‐case *R* ^2^_mean_ among the evaluated methods (Figure 4d,e). For distributional agreement, scPILOT achieved the lowest context‐averaged MMD^2^ of 0.025 and the best empirical worst‐case performance (Figure 4d,g; for other metrics, see Figure S5). The patient‐specific results showed that scPILOT maintained consistently strong performance across the eight held‐out patients, although CellOT achieved comparable results in several individual prediction tasks. These findings indicate that scPILOT provided a favorable balance between average performance and robustness across heterogeneous patient contexts.

We further performed two‐sided paired Wilcoxon signed‐rank tests using the eight held‐out patients as independent paired observations after averaging across the three random seeds. For both *R* ^2^_mean_ and MMD^2^, scPILOT performed significantly better than identity mapping, scGen, biolord, and VAEGAN, whereas the differences from CellOT did not reach statistical significance (Figure 4f). Given the limited number of independent held‐out patients, these tests had limited resolution for detecting modest differences between closely performing methods. Nevertheless, the consistently strong patient‐specific results and the best empirical worst‐case performance support the robustness of scPILOT in the across‐patient benchmark.

Cell‐type‐stratified evaluation further showed that the favorable patient‐level performance of scPILOT was broadly maintained across diverse PBMC subpopulations rather than being confined to a particular cell type. Across the eight held‐out patients, scPILOT showed the strongest and most consistent overall cell‐type‐stratified performance, frequently achieving the highest or near‐highest *R* ^2^_mean_ values and ranking among the methods with the lowest MMD^2^ values across patient–cell‐type combinations, although the best‐performing method varied in several individual subgroups (Figure S6). These results support the ability of scPILOT to preserve cell‐type‐specific perturbation responses in the across‐patient setting.

### 2.5 Perturbation Prediction Across Species With scPILOT and the Baselines

**FIGURE 5:**
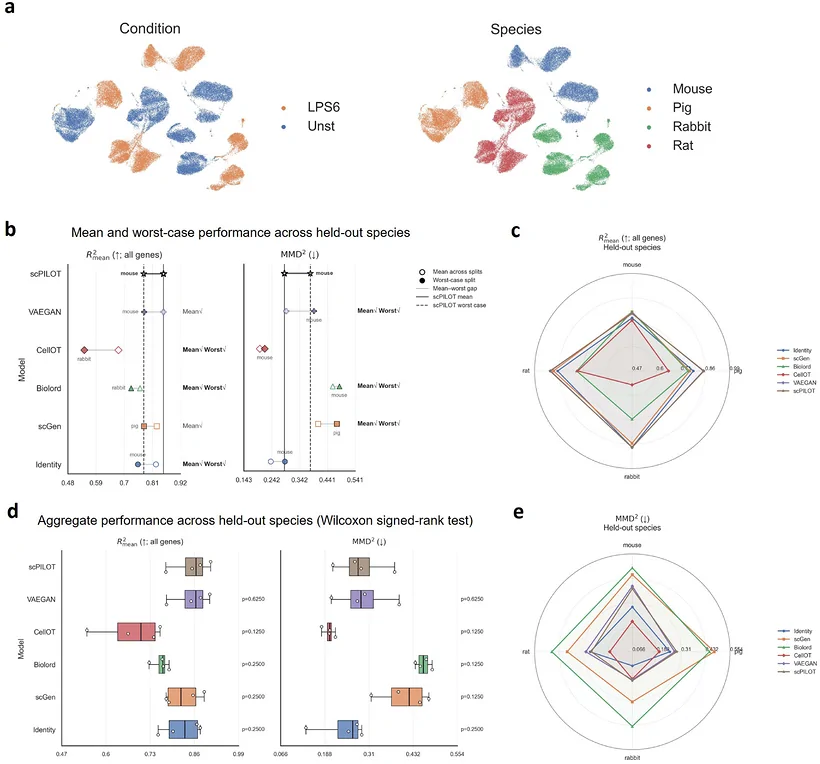
scPILOT achieves a favorable balance between prediction accuracy and distributional agreement across held‐out species. (a) UMAP visualization of the dataset comprising bone marrow‐derived mononuclear phagocytes from four species (*n* = 77,642) provided by Hagai et al. [30], labeled by condition and species. (b) Mean and empirical worst‐case *R* ^2^_mean_ and MMD^2^ across the four held‐out species. (c, e) Species‐specific *R* ^2^_mean_ and MMD^2^, respectively. (d) Aggregate performance across held‐out species and paired comparisons between scPILOT and each baseline. For (b–e), each metric was first averaged across three random seeds for each held‐out species. The *p* values in (d) were obtained using two‐sided paired Wilcoxon signed‐rank tests across the four held‐out species (*n* = 4).

Cross‐species generalization represents a challenging setting for perturbation prediction because transcriptional responses to the same stimulus may differ substantially among species, while preclinical studies frequently rely on animal models [28, 29]. We therefore evaluated scPILOT and five baseline methods using the scRNA‐seq dataset provided by Hagai et al. [30], which contains bone marrow‐derived mononuclear phagocytes from four species—mouse, pig, rabbit, and rat—under unstimulated and 6‐h lipopolysaccharide (LPS)‐stimulated conditions (Figure 5a). Each species was treated in turn as the held‐out query context: its unstimulated cells remained available for representation learning and perturbation inference, whereas its LPS‐stimulated cells were withheld and used only as the ground truth for final evaluation. Each held‐out‐species experiment was repeated using three predefined random seeds.

After averaging each metric across the three random seeds within each held‐out species, scPILOT achieved the highest context‐averaged *R* ^2^_mean_ (0.853) and maintained consistently strong predictive accuracy across the four species (Figure 5b,c). VAEGAN and scGen achieved comparable *R* ^2^_mean_ values in several species‐specific tasks, whereas CellOT showed substantially lower population‐average expression accuracy. For MMD^2^, scPILOT achieved a context‐averaged value of 0.287, lower than those of VAEGAN, scGen, and biolord but higher than those of CellOT and identity mapping (Figure 5b,e; for other metrics, see Figure S7). The low MMD^2^ of CellOT was accompanied by markedly lower *R* ^2^_mean_, indicating that its favorable distributional discrepancy did not translate into accurate recovery of population‐average gene expression.

Two‐sided paired Wilcoxon signed‐rank tests using the four held‐out species as independent paired observations did not identify statistically significant differences between scPILOT and the baseline methods for either metric (Figure 5d). Because only four independent species were available, these comparisons had limited statistical resolution. Nevertheless, the combination of the highest context‐averaged *R* ^2^_mean_ and a comparatively low MMD^2^ indicates that scPILOT provided a favorable balance between population‐average accuracy and single‐cell distributional agreement.

### 2.6 Analysis of the Relationship Between Datasets and Performance

**FIGURE 6:**
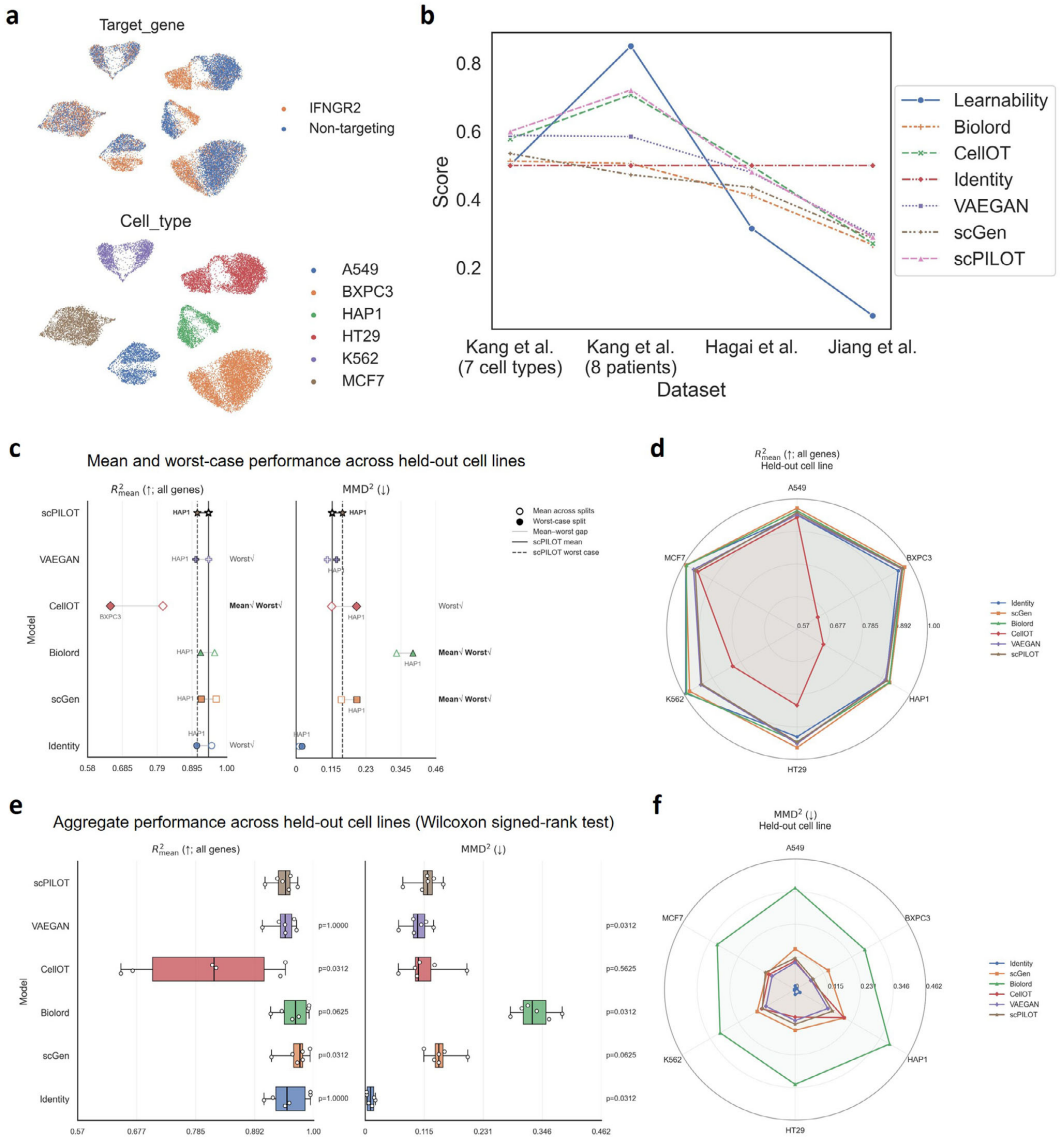
Higher dataset learnability is associated with better perturbation‐prediction performance across benchmarks. (a) UMAP visualization of the IFNGR2‐knockdown dataset comprising six cancer cell lines (*n* = 21,696) provided by Jiang et al. [31], labeled by perturbation condition and cell line. (b) Learnability scores and model performance scores across the four benchmark datasets. (c) Mean and empirical worst‐case *R* ^2^_mean_ and MMD^2^ across the six held‐out cell lines. (d, f) Cell‐line‐specific *R* ^2^_mean_ and MMD^2^, respectively. (e) Aggregate performance across held‐out cell lines and paired comparisons between scPILOT and each baseline. For (c–f) each metric was first averaged across three random seeds for each held‐out cell line. The *p* values in (e) were obtained using two‐sided paired Wilcoxon signed‐rank tests across the six held‐out cell lines (*n* = 6).

To examine how dataset characteristics relate to perturbation‐prediction performance, we additionally evaluated the models using an IFNGR2‐knockdown scRNA‐seq dataset comprising six cancer cell lines provided by Jiang et al. [31] and analyzed the results together with those from the preceding benchmarks. Each cell line was treated in turn as the held‐out query context: its non‐targeting control cells remained available for representation learning and perturbation inference, whereas the corresponding IFNGR2‐knockdown cells were withheld and used only as the ground truth for final evaluation. Each held‐out‐cell‐line experiment was repeated using three predefined random seeds. UMAP visualization showed pronounced separation among cell lines, whereas the differences between non‐targeting and IFNGR2‐knockdown cells were comparatively modest and were visually indistinct for some cell lines (Figure 6a).

After averaging each metric across the three random seeds within each held‐out cell line, scPILOT maintained high *R* ^2^_mean_ across all six prediction tasks, with context‐averaged and empirical worst‐case performance comparable to identity mapping and VAEGAN and higher than those of CellOT, biolord, and scGen (Figure 6c,d). For MMD^2^, scPILOT substantially outperformed biolord and showed performance broadly comparable to CellOT and scGen, but had higher distributional discrepancy than identity mapping and VAEGAN (Figure 6c,f; for other metrics, see Figure S8). Two‐sided paired Wilcoxon signed‐rank tests across the six held‐out cell lines supported the *R* ^2^_mean_ improvements over CellOT and scGen, whereas the differences from identity mapping and VAEGAN were not statistically significant (Figure 6e). Overall, scPILOT preserved strong population‐average expression accuracy in this benchmark, although it did not achieve the lowest distributional discrepancy. The strong performance of identity mapping, particularly for MMD^2^, suggests that IFNGR2‐knockdown‐associated changes were modest relative to the dominant transcriptional differences among cell lines.

To assess whether the relative magnitude of perturbation‐associated and biological‐context‐associated variation helps explain the differences in model performance across benchmarks, we quantified dataset learnability and a model‐level performance score combining *R* ^2^_mean_ and MMD^2^ relative to identity mapping (Methods). Higher learnability indicates that perturbation‐associated variation is larger relative to biological‐context‐associated variation, whereas identity mapping has a fixed performance score of 0.5. Across the four datasets, predictive‐model performance generally increased with dataset learnability: most predictive methods exceeded identity mapping on the higher‐learnability cell‐type and patient benchmarks, approached identity mapping on the species benchmark, and fell below identity mapping on the low‐learnability cell‐line benchmark (Figure 6b). scPILOT was among the strongest predictive methods on the higher‐learnability benchmarks, but its advantage over identity mapping diminished as learnability decreased. These results suggest that cross‐context perturbation prediction is more successful when the perturbation‐associated signal is sufficiently large relative to baseline differences among biological contexts.

The preceding analyses suggested that the relationship between the query population and the available observed contexts may influence cross‐context prediction. To examine this relationship more directly, we ranked the available observed cell‐type contexts for each held‐out query cell type according to the squared 2‐Wasserstein distance between their unperturbed latent distributions. Using the same trained checkpoints, we then compared predictions obtained using all available contexts, the three contexts with the smallest distances to the query, and the three contexts with the largest distances. Restricting inference to the three nearest contexts produced performance broadly comparable to that obtained using all available contexts across the evaluated metrics (Figure S12a), indicating that a small number of closely matched contexts captured most of the information required for response transfer. In contrast, using only the three farthest contexts generally decreased *R* ^2^_mean_ and increased MMD^2^. The paired comparison between the nearest and farthest settings confirmed this trend across most held‐out cell types (Figure S12b). Moreover, larger increases in query–context mismatch were accompanied by more pronounced performance degradation for several query cell types (Figure S12c). These results suggest that prediction performance depends more strongly on the relevance of the available observed contexts to the query population than on the total number of contexts used for inference.

### 2.7 Component‐Wise Ablation Studies of scPILOT

To clarify the actual role of each component in the final performance, we conducted component‐wise ablation studies of scPILOT on the four benchmarks across cell types, patients, species, and cell lines. We compared scPILOT with four variants obtained by separately removing the discriminator, latent OT, Leiden clustering, and adaptive weighting. The no‐discriminator model was obtained by removing the discriminator module and its associated losses and was trained independently, whereas the other three ablations were evaluated at the inference stage using the corresponding trained scPILOT model. Specifically, latent OT was replaced by mean‐shift transfer and centroid‐based matching, Leiden clustering was removed to yield global *Q*–*ρ*_U_ transfer, and adaptive weighting was replaced by equal weighting. As before, we evaluated the performance using *R* ^2^_mean_ and MMD^2^.

**FIGURE 7:**
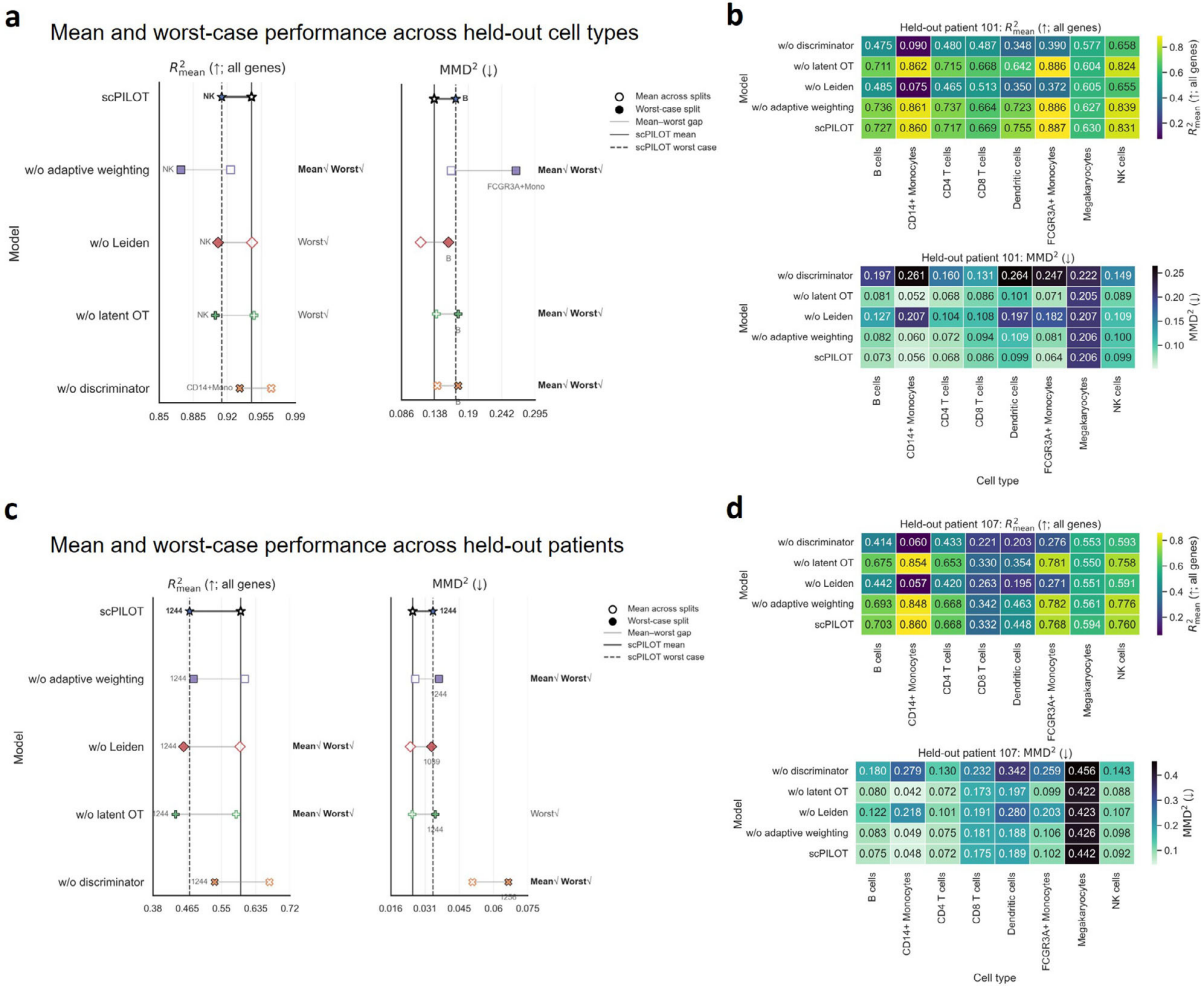
Ablation studies reveal distinct contributions of scPILOT components. (a, c) Context‐averaged and empirical worst‐case performance of scPILOT and its ablated variants across cell types and patients, respectively. Values indicating poorer performance than the complete scPILOT model are annotated. (b, d) Heatmaps showing the cell‐type‐stratified performance of the complete scPILOT model and its ablated variants for held‐out patient 101 and 107, respectively.

Figure 7a,c and Figure S9a,c show the context‐averaged and empirical worst‐case performance of scPILOT and its ablated variants across the four benchmarks. The corresponding query‐level performance profiles are provided in Figure S10. Radar plots show the results for individual held‐out queries, while boxplots summarize their distributions across queries and provide paired hypothesis tests between scPILOT and the corresponding ablated variants. Without the discriminator, *R* ^2^_mean_ increases but MMD^2^ also increases markedly, which suggests the discriminator introduces a trade‐off between single‐cell distributional fidelity and population‐average expression accuracy, improving the former while slightly compromising the latter. Moreover, we evaluated the across‐patient predictions separately for each cell type and found that the no‐discriminator model performed substantially worse (Figure 7b,d, and Figure S9b,d–h), suggesting that the discriminator helps capture cell‐type‐specific heterogeneity in perturbation responses.

To further examine the effect of discriminator‐assisted training, we compared the training and validation reconstruction MMD^2^ curves of scPILOT and the no‐discriminator variant on two representative benchmark settings with three predefined random seeds (Figure S11). Both models converged stably, but the no‐discriminator model consistently attained lower training reconstruction MMD^2^ and higher validation reconstruction MMD^2^. Together with its improved population‐average accuracy but poorer downstream distributional agreement, this enlarged train–validation discrepancy suggests objective‐specific overfitting toward the pointwise reconstruction objective. Such overfitting may manifest as oversmoothing of predicted responses toward population‐average expression patterns, thereby weakening the preservation of single‐cell and cell‐type‐specific heterogeneity. Discriminator‐assisted feature matching may mitigate this imbalance by providing an additional distribution‐sensitive constraint.

The contribution of latent OT was context dependent. Its removal produced the clearest deterioration in the across‐patient benchmark, and modestly worsened MMD^2^ in the across‐cell‐type benchmark. However, comparable or improved results were observed in some other settings, indicating that the benefit of cell‐level latent transport depends on the structure of the biological context. Leiden clustering had a limited effect on global population‐level metrics but played a more evident role in preserving local response specificity. Removing Leiden clustering produced only modest changes in aggregate *R* ^2^_mean_ and MMD^2^, with slight deterioration in some settings. However, cell‐type‐stratified evaluation in the across‐patient benchmark revealed markedly poorer performance for the no‐Leiden clustering variant, suggesting that Leiden clustering helps preserve cell‐type‐specific heterogeneity in perturbation responses. In most settings, removing adaptive weighting simultaneously reduced *R* ^2^_mean_ and increased MMD^2^, suggesting that this module contributes to both population‐average expression accuracy and single‐cell distributional fidelity.

## 3 Discussion

Single‐cell perturbation prediction across biological contexts requires not only estimating heterogeneous perturbation responses from observed unperturbed and perturbed populations, but also determining how these responses should be assigned to cells in a held‐out query context. The central methodological contribution of scPILOT lies in its query‐conditioned two‐stage latent response‐transfer procedure. In the first stage, latent OT estimates cell‐level perturbation responses from observed control–perturbed populations. In the second stage, these responses are transferred to individual query cells according to the latent‐state relationships between *Q* and *ρ*_U_. Leiden clustering localizes this transfer within transcriptionally coherent neighbourhoods, whereas adaptive weighting integrates response estimates from multiple observed populations according to their query‐specific transport costs. This formulation separates perturbation‐response estimation from localized query‐specific transfer and multi‐population integration, rather than relying on a single population‐level shift or a global control‐to‐perturbed mapping.

Within this framework, discriminator‐assisted feature matching shapes the latent representation used for subsequent response estimation and transfer. In the absence of the discriminator, optimization relies more heavily on the pointwise reconstruction objective, which may overemphasize population‐average expression patterns at the expense of heterogeneous single‐cell states. Consistent with this interpretation, the no‐discriminator variant achieved higher population‐average expression accuracy but poorer single‐cell distributional agreement, together with markedly degraded cell‐type‐stratified performance in the across‐patient benchmark. The training curves further showed that the no‐discriminator model attained lower training reconstruction MMD^2^ but consistently higher validation reconstruction MMD^2^. We interpret this pattern as objective‐specific overfitting toward pointwise reconstruction, which may manifest as oversmoothing of predicted responses and reduced preservation of single‐cell and cell‐type‐specific heterogeneity. Discriminator‐assisted feature matching helps mitigate this imbalance by introducing an additional distribution‐sensitive constraint.

The component‐wise ablations supported this functional interpretation, although the effects were metric and context dependent. The discriminator primarily improved single‐cell distributional fidelity and cell‐type‐specific response preservation, accompanied by a trade‐off in population‐average expression accuracy. The contribution of latent OT was most evident in the across‐patient benchmark, suggesting that cell‐level transport may be particularly useful when perturbation responses are transferred across heterogeneous patient contexts. Leiden clustering had only a limited effect on pooled population‐level metrics but substantially improved cell‐type‐stratified predictions, indicating that aggregate evaluations may obscure errors within individual cellular subpopulations. Adaptive weighting generally improved both population‐average expression accuracy and single‐cell distributional fidelity, supporting its role in integrating multiple response estimates according to their relevance to the query population.

scPILOT has two main limitations. First, scPILOT transfers perturbation responses from observed populations to the query population, and its performance therefore depends on whether the available observed unperturbed populations adequately represent the cellular states present in the query population. When the query cells differ substantially from all available observed unperturbed cells, the estimated perturbation responses may not transfer reliably. Our mismatch analysis confirmed that prediction performance deteriorated when only highly dissimilar observed populations were available. At the same time, the rapidly expanding scale and diversity of single‐cell perturbation datasets are likely to increase the availability of observed populations with cellular states related to those of a given query. This trend may broaden the range of query contexts for which informative response‐transfer sources can be identified, thereby improving the practical applicability of scPILOT. Nevertheless, performance may remain limited for rare or poorly represented cellular states. Second, scPILOT currently addresses context generalization for previously observed perturbations rather than perturbation‐identity generalization. Because perturbation effects are inferred from observed control–perturbed populations, the current framework cannot directly predict a perturbation that has not been observed in any biological context. Incorporating perturbation representations derived from molecular structures, gene‐function knowledge, or other prior information may extend the framework to unseen perturbations.

Future work could extend scPILOT by incorporating multimodal cellular measurements and representations learned from single‐cell foundation models. Perturbation embeddings derived from molecular structures, functional annotations, or large‐scale perturbation datasets may further enable joint generalization across both biological contexts and perturbation identities.

In conclusion, scPILOT provides a query‐conditioned approach for transferring heterogeneous responses to previously observed perturbations across biological contexts. Discriminator‐assisted feature matching further helps mitigate objective‐specific overfitting toward pointwise reconstruction and may alleviate the associated oversmoothing of single‐cell response distributions. By separating cell‐level response estimation from localized query‐specific response transfer, the framework predicts perturbed states according to the baseline‐state relationships between observed and query populations. Our results support the utility of this formulation across held‐out cell‐type, patient, species, and cell‐line settings when unperturbed query cells and sufficiently informative observed populations are available.

## 4 Methods

### 4.1 Study Design

The objective of this study was to develop and evaluate scPILOT, a single‐cell perturbation prediction framework designed for generalization across biological contexts. The central task was to predict transcriptional responses to perturbations in held‐out biological contexts, including unseen cell types, patients, species, and cell lines. scPILOT was designed as a perturbation‐type‐agnostic framework. Rather than imposing perturbation‐specific mechanistic assumptions, it learns perturbation effects from observed pre‐ and post‐perturbation single‐cell profiles, allowing the same modeling strategy to be applied to diverse perturbation modalities when appropriate control and perturbed data are available.

The study consisted of model development and a prespecified multi‐benchmark evaluation. We first formulated scPILOT as a query‐conditioned framework for predicting responses to previously observed perturbations across held‐out biological contexts, separating generative representation learning from a two‐stage latent response‐transfer procedure. During training, a variational autoencoder with discriminator‐assisted feature matching learns a generative latent representation of the observed cellular states. During inference, the learned neural‐network parameters are fixed: latent optimal transport first estimates heterogeneous cell‐level perturbation‐response vectors from observed unperturbed–perturbed populations, after which Leiden‐based localization, query‐to‐reference optimal transport, and adaptive weighting transfer and integrate these responses for the unperturbed query population. We then evaluated scPILOT across four biologically distinct held‐out‐context prediction settings. These included held‐out cell‐type prediction using the Kang et al. PBMC dataset with IFN‐β stimulation across seven cell types [18], held‐out patient prediction using the Kang et al. dataset across eight patients [18], held‐out species prediction using the Hagai et al. dataset with LPS stimulation across four species [30], and held‐out cancer cell line prediction using the Jiang et al. dataset with IFNGR2 gene knockdown across six cell lines [31].

For each benchmark, one biological context *q* was treated as the unseen query context. The perturbed cells from *q* were completely excluded from training, validation, early stopping, checkpoint selection, hyperparameter tuning, and perturbation inference, and were used only as the ground truth for final evaluation. The unperturbed cells from *q* remained available for generative representation learning and were supplied as the query population *Q* during inference. Training and validation partitions were constructed exclusively from the data visible under this protocol. Context labels were used only to define the benchmark split and organize the query and observed populations, and were not supplied as input features to the encoder, generator, or discriminator. A stage‐wise summary of data accessibility is provided in Table S1.

scPILOT was compared with scGen [11], CellOT [12], biolord [14], VAEGAN, and identity mapping. The same context‐level partitions and visible data were used for all methods. When a method required validation or early stopping, validation data were selected only from the visible training contexts according to the method‐specific protocol. Held‐out test contexts were not used for hyperparameter tuning, checkpoint selection, early stopping, or any other model‐selection procedure. The primary evaluation outcomes were *R* ^2^_mean_ and MMD^2^. Secondary outcomes included *R* ^2^_var_, *L* ^2^_mean_, and *L* ^2^_var_. As a prespecified exploratory component, we further quantified dataset learnability and a model‐level comprehensive score to examine how the relative magnitude of perturbation‐induced and context‐associated variation was associated with prediction performance across benchmarks.

### 4.2 Generative Representation Learning With Discriminator‐Assisted Feature Matching

scPILOT learns a generative latent representation using a VAE encoder–decoder optimized with reconstruction and KL‐divergence objectives, together with discriminator‐assisted feature‐matching and classification losses. The KL‐divergence objective regularizes the approximate posterior *q_ϕ_* (*z* | *x*) toward the standard normal prior *p*(*z*) = N(0, *I*):

(1)

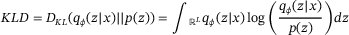

where *q_ϕ_* (*z* | *x*) denotes the approximate posterior produced by the encoder and *p*(*z*) denotes the standard normal prior.

Then, the reconstruction loss causes cells output by the VAE to approach the input:

(2) $$L_{\mathrm{reconstruction}}=\sum_{i=1}^{n}{\left(x_{i}-{\hat{x}}_{i}\right)}^{2}$$

where *x* = (*x*_1_, *x*_2_, …, *x_n_*) denotes the input and $\hat{x}=\left({\hat{x}}_{1},\;{\hat{x}}_{2},\text{…},\;{\hat{x}}_{n}\right)$ denotes the output.

Regarding the discriminator, we randomly sample the same number of latent vectors that are subject to a standard normal distribution as the cells input into the VAE and then input them into the generator. Afterwards, we input both the input and output of the VAE into the discriminator and use a layer of the discriminator to characterize the reconstruction loss and the difference between the mean of the input and output from the random latent vectors:

(3)

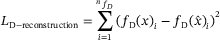

(4) $$L_{D-\mathrm{difference}}=\sum_{i=1}^{nf_{D}}{\left(\mathrm{mean}{\left(f_{D}\left(x\right)\right)}_{i}-\mathrm{mean}{\left(f_{D}\left({\hat{x}}_{p}\right)\right)}_{i}\right)}^{2}$$

where $x_{p}=\left(x_{p_{1}},x_{p_{2}},\text{…}x_{p_{n}}\right)$ denotes the output from the random latent vectors.

Then, a binary cross‐entropy (BCE) loss is used to help the discriminator classify real or fake cells more accurately:

(5) $$\begin{array}{c} L_{\mathrm{classification}}=-\left(log\left[D\left(x\right)\right]+log\left[1-D\left(\hat{x}\right)\right]+\log\left[1-D({\hat{x}}_{p}\right)\right]) \end{array}$$

Finally, we have the loss function for the VAE and the discriminator:

(6) $$\begin{array}{c} L_{\mathrm{VAE}}=\lambda_{1}KLD+\lambda_{2}\left(L_{\mathrm{reconstruction}}+L_{D-\mathrm{reconstruction}}\right)+\lambda_{3}L_{D-\mathrm{difference}} \end{array}$$

(7) $$L_{D}=\lambda_{4}L_{\mathrm{classification}}$$

During each training step, we first update the parameters of the VAE to minimize ℒ_VAE_. Then, we update the parameters of the discriminator to minimize ℒ_D_. The VAE and discriminator parameters were optimized alternately using 𝓛_VAE_ and 𝓛_D_, respectively. By minimizing the loss function, the generative networks become able to extract the latent features of a cell and reconstruct it.

### 4.3 Perturbation Inference Through Two‐Stage Latent Response Transfer

During inference, the trained encoder and generator are fixed. The inputs consist of an unperturbed query population *Q* and observed populations containing both unperturbed cells *ρ*_U_ and their corresponding perturbed cells *ρ*_P_. All cells are first encoded into the latent space. scPILOT then performs perturbation inference in two stages: Stage I estimates heterogeneous cell‐level perturbation responses from the observed populations, and Stage II transfers and integrates these responses for the query cells. The resulting query‐specific response vectors *δ'* are added to the latent representations of *Q*, and the shifted latent states are decoded to obtain the predicted perturbed expression profiles.

#### 4.3.1 Stage I: Cell‐Level Perturbation‐Response Estimation From Observed Populations

Step 1: Computation of perturbation‐response vectors *δ* for cells in *ρ*_U_

For each observed cell group in *ρ*_U_, we compute an OT coupling between its unperturbed population *ρ*_U_ and corresponding perturbed population *ρ*_P_ using POT [32]. The coupling is used to obtain the barycentric perturbed state corresponding to each unperturbed cell. The cell‐level perturbation‐response vector is defined as the difference between the transported perturbed latent state and the original unperturbed latent state: 
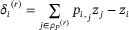
 (*i*∈*ρ*_U_^(^ *^r^* ^)^, *z* denotes latent vectors. *p* denotes the elements in the OT mapping.).

#### 4.3.2 Stage II: Query‐Specific Response Transfer

Step 2: Leiden clustering of *ρ*_U_ and *Q*

We apply Leiden [17] clustering separately to the query population *Q* and the observed unperturbed population *ρ*_U_, thereby partitioning them into smaller, transcriptionally coherent clusters. Each query cluster is paired with every cluster from *ρ*_U_ for subsequent local response transfer.

Step 3: Computation of adaptive weights for the *ρ*_U_ clusters

For each query cluster *i*, we compute the squared 2‐Wasserstein distance *d_i_*_,_ *_j_* to each observed unperturbed cluster *j*. These distances are converted into adaptive weights *w_i_*_,_ *_j_* using a softmax transformation, such that clusters with lower *Q*–*ρ*_U_ transport costs contribute more strongly to the final response estimate:

$$w_{i,j}=\frac{\exp(-d_{i,j})}{\sum_{cluster_{k}\in \rho_{U}}\exp(-d_{i,k})}$$

Step 4: Local response transfer and estimation of *δ'*

For each *Q*–*ρ*_U_ cluster pair, we compute a local OT coupling between the query cells and the observed unperturbed cells. This coupling transfers the cell‐level response vectors *δ* estimated in Step 1 to the query cells. For each query cluster, the transferred response estimates from the available *ρ*_U_ clusters are integrated using the adaptive weights obtained in Step 3, yielding the query‐specific response vectors *δ'*: 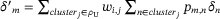 (*m*∈*cluster_i_*). The predicted perturbed latent states are then obtained by adding *δ'* to the latent representations of the query cells and are decoded by the generator to produce the final counterfactual predictions.

### 4.4 Datasets and Preprocessing

The PBMC dataset provided by Kang et al. [18] for perturbation prediction across cell types was inherited from Lotfollahi et al. [11] and the dataset containing different patients provided by Kang et al. [18] was inherited from Bunne et al. [12]. The four‐species dataset provided by Hagai et al. [30] was inherited from Lotfollahi et al. [11]. These datasets were preprocessed by the authors from which we obtained the datasets, and we would like to thank the authors for providing them. We extracted non‐targeting and IFNGR2 knockdown cells from a cancer cell line dataset of the IFNG pathway provided by Jiang et al. [31]. Afterwards, we preprocessed the data with the scanpy functions normalize_total and log1p.

### 4.5 Evaluation Metrics

Since we lacked access to the mapping relations between unperturbed and perturbed cells, we evaluated the performances of the models with 5 metrics, i.e., *R* ^2^_mean_, *L* ^2^_mean_, MMD^2^, *R* ^2^_var_, and *L* ^2^_var_.

*R* ^2^_mean_ refers to the squared Pearson's correlation coefficient between the mean gene expression of the predicted and perturbed cells. Similarly, *L* ^2^_mean_ refers to the *L* ^2^‐distance between the mean gene expression of the predicted and perturbed cells. These two metrics aim to measure the consistency of the prediction and ground truth. *R* ^2^_mean_ is more highly recommended because *L* ^2^_mean_ is unable to distinguish between inconsistent genes, and its value depends on the range of gene expression, which may differ greatly due to differences in data preprocessing; thus, we cannot judge a result from a single value but can only compare multiple values to decide which is better.

To quantify the distributional discrepancy between the predicted and observed perturbed cells, we used the squared maximum mean discrepancy [20], MMD^2^. Let *P*_pred_ and *P*_pert_ denote the predicted and observed perturbed cell distributions, respectively. For an RBF kernel

$$k_{\gamma }\left(u,v\right)=exp\left(-\gamma ∥u-v{{∥}_{2}}^{2}\right)$$

The population quantity is defined as

(8)

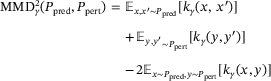

where *x* and *x'* are independent draws from *P*_pred_, and *y* and *y'* are independent draws from *P*_pert_. We used 50 inverse‐bandwidth parameters logarithmically spaced from 10^1^ to 10^−3^,

$$\gamma_{s}=10^{1-4\frac{s-1}{49}},s=1,\text{…},50$$

and reported the arithmetic mean of the 50 resulting $\mathrm{MM}{D^{2}}_{\gamma_{s}}$ values. For each *γ_s_*, the empirical quantity was computed using the biased V‐statistic:

(9)

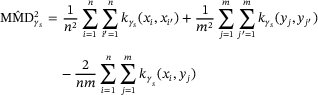




Thus, the reported metric was

(10) $$M\hat{M}D^{2}=\frac{1}{50}\sum_{s=1}^{50}M\hat{M}D_{\gamma_{s}}^{2}$$

Lower values indicate closer agreement between the predicted and observed single‐cell distributions.*R* ^2^_var_ refers to the squared Pearson's correlation coefficient between the variance in gene expression of the predicted and perturbed cells. Similarly, *L* ^2^_var_ refers to the *L* ^2^‐distance between the variance in gene expression of the predicted and perturbed cells. Among the three metrics for measuring the distribution distances/consistency, MMD^2^ is the most highly recommended because *R* ^2^_var_ and *L* ^2^_var_ would fail in an exceptional case in which the variances are very close but the values are completely different, e.g., two parallel distributions.

### 4.6 Feature Selection

The *R* ^2^ and *L* ^2^ values were computed by using all genes. MMD^2^ was computed by using the top 50 DEGs because the computation of MMD^2^ is biased with high scRNA‐seq data dimensionality [12]. The DEGs were computed with the scanpy [21] function rank_genes_groups by using unperturbed and perturbed cells from specific population, and for UMAP [19] visualization, we first carried out a principal component analysis (PCA) producing 50 PCs, then selected significant PCs with the scanpy [21] function pca_variance_ratio, and finally executed UMAP with the significant PCs.

### 4.7 Dataset Learnability Analysis

We performed a prespecified exploratory analysis to quantify the learnability of each benchmark dataset for cross‐biological‐context perturbation prediction. The rationale was that a dataset should be more learnable when perturbation‐induced transcriptional variation is large relative to biological‐context‐associated variation. In such cases, perturbation effects are expected to be more transferable from observed contexts to an unseen context.

For each dataset, let ${{\bar{x}}_{i}}^{\mathrm{per}}$ and ${{\bar{x}}_{i}}^{\mathrm{unp}}$ denote the mean perturbed and unperturbed expression vectors in the *i*‐th biological context, respectively, and let ${{\bar{x}}_{-i}}^{\mathrm{unp}}$ denote the mean unperturbed expression vector across all other contexts. Dataset learnability was defined as the average normalized perturbation‐to‐context variation ratio across all *K* contexts:

(11) $$Learnability=\frac{1}{K}\sum_{i=1}^{K}\frac{\left|\right|{{\bar{x}}_{i}}^{\mathrm{per}}-{{\bar{x}}_{i}}^{\mathrm{unp}}|{{|}_{2}}^{2}}{\left|\right|{{\bar{x}}_{i}}^{\mathrm{per}}-{{\bar{x}}_{i}}^{\mathrm{unp}}|{{|}_{2}}^{2}+\left|\right|{{\bar{x}}_{-i}}^{\mathrm{unp}}-{{\bar{x}}_{i}}^{\mathrm{unp}}|{{|}_{2}}^{2}}$$

This score ranges from 0 to 1. A higher value indicates that perturbation‐associated variation is larger relative to context‐associated variation in the unperturbed state, suggesting that the perturbation response is more identifiable and potentially more transferable across biological contexts. Conversely, a lower value indicates that context‐associated variation in the unperturbed state is larger, suggesting a more challenging prediction setting.

### 4.8 Model‐Level Comprehensive Score

To summarize model performance, we further defined a model‐level comprehensive score using identity mapping as the baseline. For each model and benchmark, *R* ^2^_mean_ and MMD^2^ were first averaged across the three random seeds within each held‐out biological context. The resulting context‐level values were then averaged equally across all held‐out contexts in the benchmark to obtain the model‐level mean *R* ^2^_mean_:

(12) $${{\bar{R}}^{2}}_{\mathrm{mean},m}=\frac{1}{K}\sum_{q=1}^{K}\left(\frac{1}{3}\sum_{s=1}^{3}R_{\mathrm{mean},\;m,q,s}^{2}\right)$$

and MMD^2^:

(13) $$M\bar{M}D_{\mathrm{mean},m}^{2}=\frac{1}{K}\sum_{q=1}^{K}\left(\frac{1}{3}\sum_{s=1}^{3}\mathrm{MM}D_{\mathrm{mean},\;m,q,s}^{2}\right)$$

For model *m*, the score was computed as:

(14) $$\begin{array}{c} Score_{m}=\frac{1}{2}\left(\frac{{{\bar{R}}^{2}}_{\mathrm{mean},m}}{{{\bar{R}}^{2}}_{\mathrm{mean},m}+{{\bar{R}}^{2}}_{\mathrm{mean},\mathrm{identity}}}+\frac{M\bar{M}D_{\mathrm{identity}}^{2}}{M\bar{M}D_{\mathrm{identity}}^{2}+M\bar{M}D_{m}^{2}}\right) \end{array}$$

where *m* denotes model, *K* denotes the number of contexts, *q* denotes query, *s* denotes seed, and the overline denotes the average metric value across held‐out contexts. This score combines the relative improvement in *R* ^2^_mean_, for which higher values indicate better performance, and MMD^2^, for which lower values indicate better performance. A score greater than 0.5 indicates better overall performance than identity mapping, whereas a score below 0.5 indicates worse overall performance. This analysis was used to interpret the relationship between dataset learnability and model performance and was not treated as a primary endpoint for model comparison.

### 4.9 Query–Context Mismatch Analysis

To assess the sensitivity of response transfer to query–context mismatch, we performed an inference‐only analysis on the across‐cell‐type benchmark. For each held‐out query cell type and model‐initialization seed, the available observed cell‐type contexts were ranked according to the empirical squared 2‐Wasserstein distance between their unperturbed latent distributions and that of the query population. Using the same trained checkpoint, predictions were generated using all available observed contexts, the three nearest contexts, or the three farthest contexts. For each selected context set, the mean mismatch score was calculated as the arithmetic mean of the query–context squared 2‐Wasserstein distances across the included observed contexts. No model retraining, checkpoint reselection, or hyperparameter adjustment was performed. Prediction performance was evaluated using *R* ^2^_mean_ and MMD^2^, and the results were averaged across the three model‐initialization seeds.

### 4.10 Statistical Analysis

Statistical analyses were performed at the level of independent held‐out biological contexts. For each benchmark and model, each evaluation metric was first averaged across the three random seeds within each held‐out context. Pairwise comparisons between scPILOT and each baseline or ablated variant were then conducted across held‐out contexts using two‐sided paired Wilcoxon signed‐rank tests. The independent paired observations were the seven held‐out cell types, eight held‐out patients, four held‐out species, or six held‐out cell lines, depending on the benchmark. Individual‐context results were interpreted descriptively, whereas benchmark‐level *p* values were reported in the corresponding figures and captions.

### 4.11 Component‐Wise Ablation Settings

The no‐discriminator variant was trained independently after removing the discriminator and all discriminator‐related loss terms. The other three ablations were implemented only at inference using the same trained scPILOT. In the no‐latent‐OT variant, perturbation effects of observed populations were represented by mean latent shifts, and *Q*–*ρ*_U_ matching was performed using centroid distances. In the no‐Leiden‐clustering variant, Leiden clustering was omitted and *Q*–*ρ*_U_ response transfer was performed globally. In the no‐adaptive‐weighting variant, OT‐cost‐based adaptive weights were replaced with equal weights across all *ρ*_U_ estimates. All other settings were kept unchanged.

## Author Contributions

X.L., Z.D. and Y.L. supervised the study. J.W., Z.L., Z.Z. and Y.L. conceived and designed the study. J.W., X.L., Z.D. and Y.L. developed the algorithm. J.W., Z.L. and Z.Z. collected and curated the data and conducted the experiments. J.W., Z.L., Z.Z., X.L., Z.D. and Y.L. verified the reliability of the results. J.W., J.R., P.C., J.T., L.X., X.L., Z.D. and Y.L. analysed the results. J.W., Z.D. and Y.L. wrote the manuscript. All authors reviewed and approved the final manuscript.

## Funding

This work was supported by Science and Technology Innovation 2030 – “Cancer, Cardiovascular and Cerebrovascular, Respiratory and Metabolic Diseases Prevention and Treatment Research” Major Project (Project No. 2023ZD0506403) by Medical and Health Science and Technology Development Research Center, National Health Commission of People's Republic of China and Heilongjiang Provincial Science and Technology Department (No: 2022ZX103C01).

## Conflicts of Interest

The authors declare no conflicts of interest.

## Code Availability

The code of scPILOT and the scripts for reproducing the results are available at https://github.com/yongzhuangliulab/scPILOT.

## Supporting information




**Supporting File**: advs77461‐sup‐0001‐SuppMat.docx.

## Data Availability

The data that support the findings of this study are openly available in Zenodo at https://zenodo.org/records/17827977, reference number 17827977.
