# Supplementary material for: Predicting Single‐Cell Perturbation Responses Across Biological Contexts With a Deep Generative Model Integrating Optimal Transport
Source: Adv Sci (Weinh). 2026 Aug 29:e77461. Online ahead of print. doi: 10.1002/advs.77461 (PMC13525497; doi:10.1002/advs.77461)
Supplement: Supplementary file 1 [file ADVS-9999-e77461-s001.docx]

SUPPLEMENTARY MATERIALS

**Supplementary notes**

| **Algorithm 1:** Generative Representation Learning Framework of scPILOT | |
| --- | --- |
| **Input:** Visible single-cell expression profiles *x* ∈ ℝ*^n^*^×^*^d^*; mini-batch size *B*; latent dimension *L*; loss coefficients *λ*_1_, *λ*_2_, *λ*_3_, *λ*_4_; maximum number of epochs *ξ*.  **Output:** Trained encoder *E_ϕ_*, generator *G_θ_*, and discriminator *D_ω_*. | |
| 1 | **Initialization:** |
| 2 | Initialize model parameters *ϕ*, *θ*, and *ω*; |
| 3 | **for** *e* ← 1 **to** *ξ* **do** |
| 4 | **for each** mini-batch {*x_i_*}*^b^_i_*_=1_, where *b* ≤ *B*, **do** |
| 5 | **Forward Pass:** |
| 6 | Compute the posterior parameters and sample the latent representations: |
|  | *μ_i_*, *v_i_* ← *E_ϕ_*(*x_i_*), *z_i_* ~ *q_ϕ_*(*z* \| *x_i_*) = N(*μ_i_*, diag(*v_i_*)); |
| 7 | Reconstruct the input cells: |
|  | $\hat{x}_{i}\leftarrow G_{\theta}(z_{i})$; |
| 8 | Sample latent vectors from the standard normal prior and generate additional synthetic cells: |
|  | *z_p_*_,_*_i_* ~ *p*(*z*) = N(0, *I_L_*), $\hat{x}_{p,i}\leftarrow G_{\theta}(z_{p,i})$; |
| 9 | Compute the intermediate discriminator representations: |
|  | *f_D_*(*x_i_*), $f_{D}(\hat{x}_{i})$, $f_{D}(\hat{x}_{p,i})$; |
| 10 | Compute the discriminator probabilities: |
|  | *s_i_* ← *D_ω_*(*x_i_*), $\hat{s}_{i}\leftarrow D_{\omega}(\mathrm{sg}(\hat{x}_{i}))$, $\hat{s}_{p,i}\leftarrow D_{\omega}(\mathrm{sg}(\hat{x}_{p,i}))$; |
| 11 | **Loss Computation:** |
| 12 | Compute the posterior-to-prior KL divergence for each cell: |
|  | ; |
| 13 | Compute the VAE objective: |
|  | ; |
| 14 | Compute the discriminator objective: |
|  | ; |
| 15 | **Update Model Parameters:** |
| 16 | Update the encoder and generator parameters while keeping the discriminator parameters fixed: |
|  | (*ϕ*, *θ*) ← (*ϕ*, *θ*) − *η*_VAE_ ∇*_ϕ_*_,_*_θ_*ℒ_VAE_; |
| 17 | Update the discriminator parameters while keeping the encoder and generator parameters fixed: |
|  | *ω* ← *ω* − *η_D_* ∇*_ω_*ℒ_D_; |
| 18 | **end** |
| 19 | **end** |
| 20 | **Output:** Trained encoder *E_ϕ_*, generator *G_θ_*, and discriminator *D_ω_*. |

Here, *n* denotes the number of visible cells, *d* denotes the number of genes, *B* denotes the maximum mini-batch size, and *L* denotes the latent-space dimensionality. The vector *v_i_* contains the posterior variances, *f_D_*(·) denotes the intermediate discriminator representation used for feature matching, sg(·) denotes the stop-gradient operation, and *ε* is a small numerical-stability constant.

| **Algorithm 2:** Query-Conditioned Two-Stage Latent Response Transfer of scPILOT | |
| --- | --- |
| **Input:** Trained encoder *E_ϕ_* and generator *G_θ_*; unperturbed query population *Q*; observed unperturbed and perturbed population pairs {(*ρ*_U_^(^*^r^*^)^, *ρ*_P_^(^*^r^*^)^)}^R^*_r_*_=1_; latent dimension *L*; numerical-stability constant *ε*.  **Output:** Predicted perturbed expression profiles $\hat{Q}_{\text{P}}$. | |
| 1 | **Initialization:** |
| 2 | Fix the trained parameters *ϕ* and *θ*; the discriminator is not used during inference; |
| 3 | Encode the query and observed populations into the latent space: |
|  | *z_Q_* ← *E_ϕ_*(*Q*), *z*_U_^(^*^r^*^)^ ← *E_ϕ_*(*ρ*_U_^(^*^r^*^)^), *z*_P_^(^*^r^*^)^ ← *E_ϕ_*(*ρ*_P_^(^*^r^*^)^); |
| 4 | **Stage I: Cell-Level Perturbation-Response Estimation** |
| 5 | **for** *r* ← 1 **to** *R* **do** |
| 6 | Compute the latent OT coupling between *ρ*_U_^(^*^r^*^)^ and *ρ*_P_^(^*^r^*^)^; |
|  | *P*^U → P,(^*^r^*^)^ = [*p_ab_*^U→P,(^*^r^*^)^] ← OT(*z*_U_^(^*^r^*^)^, *z*_P_^(^*^r^*^)^); |
| 7 | **for each** unperturbed observed cell a ∈ *ρ*_U_^(^*^r^*^)^ **do** |
| 8 | Compute its barycentric perturbed latent state and cell-level perturbation-response vector: |
|  | $\delta_{a}^{(r)}\leftarrow\frac{\sum_{b} p_{ab}^{\text{U}\to\text{P},(r)}z_{\text{P},b}^{(r)}}{\sum_{b} p_{ab}^{\text{U}\to\text{P},(r)}+\varepsilon}-z_{\text{U},a}^{(r)}$; |
| 9 | **end** |
| 10 | **end** |
| 11 | **Stage II: Query-Specific Response Transfer** |
| 12 | Apply Leiden clustering separately to the query and observed unperturbed populations: |
|  | , ; |
| 13 | **for each** query cluster *C_i_^Q^* ∈ 𝐶*_Q_* **do** |
| 14 | **for each** *C_j_*^U^ ∈ 𝐶_U_ **do** |
| 15 | Compute the local query-to-observed OT coupling and the corresponding squared 2-Wasserstein distance: |
|  | *P^Q^*^→U, (^*^i^*^,^*^j^*^)^, *d_i_*_,_*_j_* ← OT(*z_Q_*[*C_i_^Q^*], *z*_U_[*C_j_*^U^]), *P^Q^*^→U, (^*^i^*^,^*^j^*^)^ = [*p_mn_^Q^*^→U, (^*^i^*^,^*^j^*^)^]; |
| 16 | **for each** query cell *m* ∈*C_i_^Q^* **do** |
| 17 | Transfer the observed response vectors to query cell $m$: |
|  | $\tilde{\delta}_{m,j}\leftarrow\frac{\sum_{n\in C_{j}^{\text{U}}} p_{mn}^{Q\to\text{U},(i,j)}\delta_{n}}{\sum_{n\in C_{j}^{\text{U}}} p_{mn}^{Q\to\text{U},(i,j)}+\varepsilon}$; |
| 18 | **end** |
| 19 | **end** |
| 20 | Compute the adaptive weight of each observed cluster: |
|  | $w_{i,j}=\frac{\exp(-d_{i,j})}{\sum_{k=1}^{K_{\text{U}}} \exp(-d_{i,k})}$ |
| 21 | **for each** query cell *m* ∈*C_i_^Q^* **do** |
| 22 | Integrate the transferred response estimates: |
|  | $\delta_{m}^{'}\leftarrow\sum_{j=1}^{K_{\text{U}}} w_{i,j}\tilde{\delta}_{m,j}$ |
| 23 | Compute the predicted perturbed latent state: |
|  | *z*_pred,_*_m_* ← *z_Q_*_,_*_m_* + *δ'_m_*; |
| 24 | Decode the predicted perturbed expression profile: |
|  | $\hat{x}_{\text{P},m}\leftarrow G_{\theta}(z_{pred,m})$ |
| 25 | **end** |
| 26 | **end** |
| 27 | Collect all predicted perturbed expression profiles: |
|  | $\hat{Q}_{\text{P}}\leftarrow\{\hat{x}_{\text{P},m}\}_{m\in Q}$ |
| 28 | **Output:** Predicted perturbed expression profiles $\hat{Q}_{\text{P}}$. |

Here, *R* denotes the number of observed unperturbed–perturbed population pairs, *L* denotes the latent-space dimensionality, and *K_Q_* and *K*_U_ denote the numbers of Leiden clusters in the query and observed unperturbed population, respectively. *P*^U→P,(^*^r^*^)^ and *P^Q^*^→U,(^*^i^*^,^*^j^*^)^ denote the two types of latent OT couplings, and *d*_(_*_i_*_,_*_j_*_)_ denotes the squared 2-Wasserstein distance. The OT couplings, Leiden partitions, response vectors, transport distances, and adaptive weights are computed during inference and are not learned model parameters.

**Supplementary figures**

**Figure S1:** Linear regression for the mean gene expression between predicted and perturbed cells for seeds 1327, 1337, and 1347 from left to right with the top 10 DEGs highlighted (**a:** B, **b:** CD14+Mono, **c:** CD4T, **d:** CD8T, **e:** Dendritic, **f:** FCGR3A+Mono, **g:** NK. *n*_genes_=6,998, *R*^2^ denotes the squared Pearson correlation).

**Figure S2:** Integrations of the metrics (**a:** *R*^2^_var_, **b:** *L*^2^_mean_, **c:** *L*^2^_var_) in radar plots.

**Figure S3:** Marginal distributions of the top 10 DEGs for seeds 1327, 1337, and 1347 from left to right (**a:** B, **b:** CD14+Mono, **c:** CD4T, **d:** CD8T, **e:** Dendritic, **f:** FCGR3A+Mono, **g:** NK).

**Figure S4:** Marginal distributions of the top 10 DEGs for seeds 1327, 1337, and 1347 from left to right (a: 101, b: 107, c: 1015, d: 1016, e: 1039, f: 1244, g: 1256, h: 1488).

**Figure S5:** Integrations of the metrics (a: *R*^2^_var_, b: *L*^2^_mean_, c: *L*^2^_var_) in radar plots.

**Figure S6:** Heatmaps of *R*^2^_mean_ and MMD^2^ of cell-type-stratified evaluation for held-out patients (**a**–**h**, 101, 107, 1015, 1016, 1039, 1244, 1256, and 1488). Each value represents the mean across three random seeds for the corresponding patient–model–cell-type combination.

**Figure S7:** Integrations of the metrics (a: *R*^2^_var_, b: *L*^2^_mean_, c: *L*^2^_var_) in radar plots.

**Figure S8:** Integrations of the metrics (a: *R*^2^_var_, b: *L*^2^_mean_, c: *L*^2^_var_) in radar plots.

**Figure S9: a, c,** Context-averaged and empirical worst-case performance of scPILOT and its ablated variants across species and cell lines, respectively. **b, d, e-h,** Heatmaps showing the cell-type-stratified performance of the complete scPILOT model and its ablated variants for held-out patient 1015, 1016, 1039, 1244, 1256, and 1488, respectively.

**Figure S10: a, c, e, g,** Box plots summarizing the distributions of query-level performance across cell types, patients, species, and cell lines, respectively. *p* values were calculated using two-sided paired Wilcoxon signed-rank tests comparing complete scPILOT with each ablated variant. **b, d, f, h,** Radar plots showing the corresponding performance profiles for individual held-out queries in the across-cell-type, across-patient, across-species, and across-cell-line benchmarks, respectively.

**Figure S11: Discriminator-assisted training yields lower validation reconstruction MMD^2^ despite higher training reconstruction MMD^2^.** Training and validation trajectories of gene-space reconstruction MMD^2^ for the across-patient benchmark with patient 101 held out (**a, b**) and the across-species benchmark with mouse held out (**c, d**). **a** and **c** show the results from three model-initialization seeds (1327, 1337, and 1347) separately. **b** and **d** show the mean ± SD curves across the three seeds. Filled circles indicate the epochs with the lowest recorded validation reconstruction MMD^2^ for each model. Reconstruction MMD^2^ was averaged across minibatches within each epoch and was used as an auxiliary training diagnostic, distinct from the downstream perturbation-prediction MMD^2^.

**Figure S12: Closely matched observed contexts support robust cross-cell-type prediction, whereas highly mismatched contexts degrade performance. a,** Performance using all available observed contexts, the three nearest contexts, or the three farthest contexts for each held-out cell type. **b,** Paired comparison between the nearest-three and farthest-three settings. **c,** Relationship between mean query–context mismatch and prediction performance. Context mismatch was quantified by the squared 2-Wasserstein distance between unperturbed latent distributions. Results were averaged across three model-initialization seeds. Higher *R*^2^_mean_ and lower MMD^2^ indicate better performance.

**Table S1: Data accessibility during scPILOT training, inference, and evaluation**

| **Data component** | **Representation learning** | **Validation and model selection** | **Perturbation inference** | **Final evaluation** |
| --- | --- | --- | --- | --- |
| Unperturbed cells from observed contexts | Available | Available within the visible-data split | Used as *ρ*_U_ | Not used as ground truth |
| Perturbed cells from observed contexts | Available | Available within the visible-data split | Used as *ρ*_P_ | Not used as ground truth |
| Unperturbed cells from the query context | Available | Available within the visible-data split | Used as *Q* | May be shown as the baseline |
| Perturbed cells from the query context | Unavailable | Unavailable | Unavailable | Used only as ground truth |
| Context labels | Split and grouping only | Split and grouping only | Population organization only | Result stratification only |

“Available” indicates that the corresponding data are accessible at that stage, although individual cells may be assigned to either the training or validation subset according to the visible-data split.
